# Comparative susceptibility to *Plasmodium falciparum *of the molecular forms M and S of *Anopheles gambiae *and *Anopheles arabiensis*

**DOI:** 10.1186/1475-2875-10-269

**Published:** 2011-09-19

**Authors:** Mamadou O Ndiath, Anna Cohuet, Ablaye Gaye, Lassana Konate, Catherine Mazenot, Ousmane Faye, Christian Boudin, Cheikh Sokhna, Jean-François Trape

**Affiliations:** 1 Unité de Recherche sur les Maladies Infectieuses et Tropicales Emergentes (URMITE), IRD, BP 1386 Dakar, Sénégal; 2 Maladies infectieuses et vecteurs: écologie, génétique, évolution et contrôle (MIVEGEC), (UM1-CNRS 5290-IRD 224), IRD-IRSS, 01 BP 171 Bobo Dioulasso, Burkina Faso; 3 Laboratoire Ecologie Vectorielle et Parasitaire, UCAD, BP 5005 Fann Dakar, Sénégal

## Abstract

**Background:**

The different taxa belonging to *Anopheles gambiae *complex display phenotypic differences that may impact their contribution to malaria transmission. More specifically, their susceptibility to infection, resulting from a co-evolution between parasite and vector, might be different. The aim of this study was to compare the susceptibility of M and S molecular forms of *Anopheles gambiae *and *Anopheles arabiensis *to infection by *Plasmodium falciparum*.

**Methods:**

F3 progenies of *Anopheles gambiae s.l*. collected in Senegal were infected, using direct membrane feeding, with *P. falciparum *gametocyte-containing blood sampled on volunteer patients. The presence of oocysts was determined by light microscopy after 7 days, and the presence of sporozoite by ELISA after 14 days. Mosquito species and molecular forms were identified by PCR.

**Results:**

The oocyst rate was significantly higher in the molecular S form (79.07%) than in the M form (57.81%, Fisher's exact test p < 0.001) and in *Anopheles arabiensis *(55.38%, Fisher's exact test vs. S group p < 0.001). Mean ± s.e.m. number of oocyst was greater in the *An. gambiae *S form (1.72 ± 0.26) than in the *An. gambiae *M form (0.64 ± 0.04, p < 0.0001) and in the *An. arabiensis *group (0.58 ± 0.04, vs. S group, p < 0.0001). Sporozoite rate was also higher in the molecular form S (83.52%) than in form M (50.98%, Fisher's exact test p < 0.001) and *Anopheles arabiensis *50.85%, Fisher's exact test vs. S group p < 0.001).

**Conclusion:**

Infected in the same experimental conditions, the molecular form S of *An. gambiae *is more susceptible to infection by *P. falciparum *than the molecular form M of *An*. *gambiae *and *An. arabiensis*.

## Background

*Plasmodium falciparum*, the deadliest agent of human malaria, is exclusively transmitted by *Anopheles *mosquitoes. In Africa, species belonging to the *Anopheles gambiae *complex are responsible for a large proportion of malaria cases. This complex is composed of species morphologically identical but distinct in their distribution, ecology and contribution in malaria transmission. While *Anopheles merus, Anopheles melas, Anopheles bwambae *and *Anopheles quadriannulatus *have sporadic or null role in malaria transmission due to restricted geographical distribution and/or zoophily, *Anopheles gambiae s.s*. and *Anopheles arabiensis *are the most important in terms of epidemiology [1,2]. *Anopheles gambiae s.s*. itself was shown to be subdivided in incipient species, namely M and S molecular forms [3], both vectors of malaria parasites [4,5]. Although these three taxa coexist in many zones, as in Senegal [4], they have specific ecological niches and one species can be predominant on the others depending on the environmental conditions [6,7]. Especially at the larval stage, an habitat segregation has been demonstrated between M and S molecular forms [8]. The biological differences between the incipient species most likely impact their vectorial capacity and their contribution in malaria transmission [9]. A complete understanding of their role in malaria transmission would however not be possible without deciphering their relative susceptibility to the parasites.

The susceptibility of *Anopheles *mosquitoes to *Plasmodium *infection reflects the probability of successful parasite development from gamete fertilization to sporozoite production. During the sporogonic development steps in the mosquito midgut lumen, epithelium and haemolymph, parasites face a hostile environment, leading to a considerable reduction in the number of parasites reaching the oocyst stage [10-12]. The mosquito susceptibility is the result of evolutionary process on both parasite and vector that maintains susceptible and refractoriness alleles in natural populations [13]. Recently, a new cryptic sub-group inside *An. gambiae s.s*., named 'Goundry', was identified [14]. The exophilic behaviour of the taxa explains that it was not sampled before in dwellings. This new vector may have major importance in malaria transmission as it was found to be more susceptible to infection that the endophilic vectors. This finding highlighted that sibling species can have different levels of vector competence, which with regards to their vectorial capacity, will define their role in malaria transmission. Unfortunately, the study did not compare susceptibility of the already known species in the *An. gambiae *complex.

The aim of the present study was to investigate the potential difference in susceptibility of the three malaria vectors of the *An. gambiae *complex in Senegal to infection by wild isolates of *P. falciparum *from the same area, using an *in vitro *model of infection.

## Methods

### Mosquito collection

*Anopheles gambiae *s.s. larvae were collected in five different breeding sites (minimum 100 larvae per site) in the village of Dielmo (13°43'N, 16°24'W). Larvae were raised until emergence; adults were fed on rabbit blood and 100 females (F0) randomly selected (20 from each collection site). *An. arabiensis *larvae were sampled in one site in Dakar (14°72'N, 17°31'W). Larvae were also raised until emergence, fed on rabbit blood and 100 females (F0) randomly selected. Each F0 females was allowed to lays its eggs individually, before it was genotyped for the species and molecular forms by PCR-RFLP [15]. According to the experiment, among the 100 selected *An. gambiae*, 44 to 54 were molecular form M and 43 to 52 molecular form S (a few *An. arabiensis *identified were discarded). All specimens sampled in Dakar were confirmed to be *An. arabiensis*. The offspring of F0 females of the same taxa were then pooled and bred together in the same conditions for the three groups. Larvae were fed with Tetramin fish food. Pupae were collected and placed in 10-L plastic buckets, which were covered with mosquito gauze and provided with a cotton sleeve for easy access to 10% glucose on filter paper. Adults were maintained in a room at 27°C, 70% relative humidity and 12:12 h light/darkness, with a 30-mn dawn and dusk light regimen. In order to increase the proportion of mosquitoes accustomed to feeding on membrane, a selection of aggressive F1 and F2 females was performed. F3 females used for infection were all genotyped and species and molecular forms were confirmed.

### Gametocyte carriers

Gametocyte carriers were detected by cross-sectional surveys in villages and schools, during the high transmission period from September to November 2006 in Hanene, (14°47'N, 16°55'W Thies region). Informed consent was obtained from adults or legal guardians of minors. Fingerprick blood was taken from each volunteer. The thick blood smears were stained with 10% Giemsa and examined microscopically with (100×) oil immersion lens for the presence of sexual and asexual parasites. Parasite density was estimated by counting against 1,000 white blood cells and converted to numbers of parasites per μL by assuming a standard white blood cell count of 8,000/μL. Symptomatic or non symptomatic individuals having an asexual density exceeding 1,000 parasites/mm^3 ^were treated with artemisinin-based combination therapy according to national recommendations. Inclusion criteria of gametocyte carriers were: (1) age over 10 years; (2) a *P. falciparum *gametocyte density over 20/mm^3 ^of blood; and (3) no anti-malarial treatment in the previous month. 6 mL of blood were drawn from the gametocyte carriers in a heparinised vacutainer tube. An insecticide-impregnated bed net was given to the participating individuals as compensation. This study was approved by the Senegalese National Ethical Committee.

### Direct membrane feeding assay

Experimental infections were carried out by direct membrane feeding assays as described by Mulder *et al *[16]. Blood was rapidly distributed to three pools of three-days old females belonging to each taxa, through a warm-water (37°C) jacketed membrane feeder serially connected. Female mosquitoes were allowed to feed for 15 min before, partially fed and non-fed specimens were removed. Two batches of 50 mosquitoes of each taxa were randomly-selected from among fed females and maintained in the insectary under 10% sucrose diet for further analyses. The first batch of mosquitoes was dissected seven days later. Midguts were stained with 3% mercurochrome in PBS, and examined under light microscopy (40 × objective) for detection and quantification of oocysts. The percentage of oocyst-positive mosquitoes and number of oocysts were recorded. A second batch of mosquitoes were used to evaluated the presence of circumsporozoite protein (CSP) of *P. falciparum *using an enzyme-linked immunosorbent assay (ELISA)[17] performed on heads and thoraces 14 days after feeding. PCR RFLP [15] were performed on the carcasses of dissected mosquitoes and the identification of molecular forms were confirmed.

Experiments were repeated four times with different samples of gametocyte-containing blood. Gametocytaemia was 413, 574, 597 and 866 gametocytes per μL, respectively for experiments 1, 2, 3 and 4.

### Statistical analysis

Mortality rates were calculated after 7 and 14 days in each group. The number of oocyst was compared using non-parametric Kruskal-Wallis tests. Mortality rates, percentage of infected mosquito at oocyst and sporozoite stages were compared using Pearson Chi^2 ^or Fisher exact test. Correlation between the number of oocyst and the gametocytemia was analysed using Spearman test. Statistical analyses were performed using Stata^® ^10.1. A P value of 0.05 or less was considered as significant.

### Ethical approval

Experiments involving human subjects, population screenings as well as collection of blood samples, have been conducted in full accordance with ethical principles. Free and informed consent of the donors or their guardians was obtained at all times while community consent had been obtained beforehand. This study was approved by the Ethical National Comity of Senegal.

## Results

### Oocysts rates

From the 600 fed mosquitoes used for oocyst analysis, 559 survived for seven days and were included in the analysis. Mortality rates was significantly higher in *An. gambiae *S group (14%) than in the *An. arabiensis *group (2.5%) and in the *An. gambiae *M group (4%) (Fisher exact p < 0.001 and p = 0.001 respectively).

**Figure 1:**
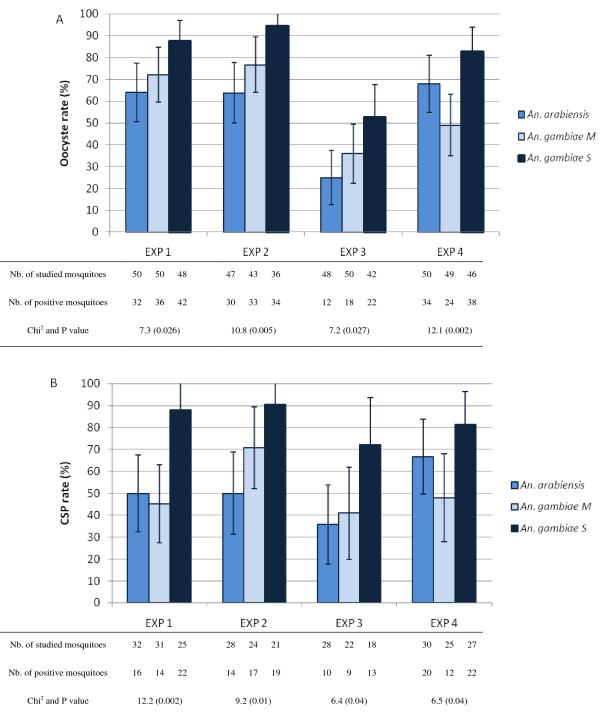
**Oocyst (Panel A) and CSP rates (Panel B) with 95% confidence interval, observed during experiments 1 to 4 (EXP 1 to EXP 4) in the three species and molecular forms**. Number of mosquitoes studied (surviving) and positive as well as Pearson Chi^2 ^and corresponding p value are give in the table.

The parasite infection rates at oocyst stage in each group are presented in the Figure 1A. In each experiment, there was a significant difference between groups (Pearson Chi^2 ^p ranging from 0.002 to 0.027) with *An. gambiae *S group being significantly more infected than *An. gambiae *M group in experiment 1 and 4 (Fisher's test p = 0.048 and 0.001 respectively), but not in experiment 2 and 3 (Fisher's test p = 0.064 and 0.086 respectively). *Anopheles gambiae *S group was significantly more infected than *An. arabiensis *group in experiment 1, 2 and 3 (Fisher's test p = 0.006, 0.001 and 0.007 respectively), but not in experiment 4 (Fisher's test p = 0.078). When analysed globally for all the four experiments, infection rate was higher in the *An. gambiae *S group (79.07%) than in the two other groups (55.38% for *An. arabiensis *and 57.81% for *An. gambiae *M respectively, Fisher's exact test p < 0.001 for each). On the other hand no significant difference was found between *An. arabiensis *and *An. gambiae *M groups (Fisher exact test p = 0.29).

**Figure 2:**
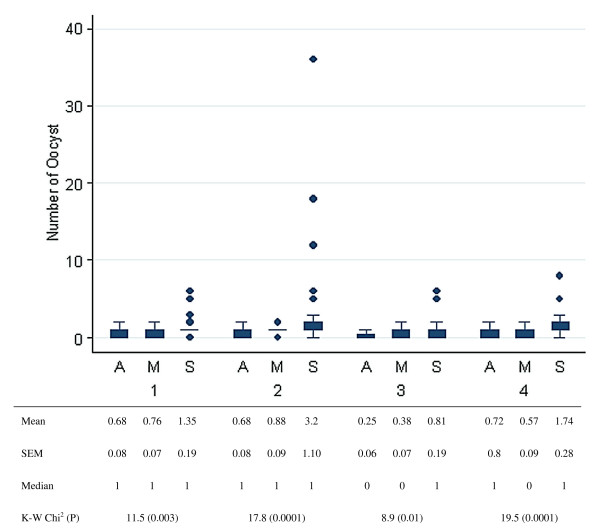
**Distribution of oocysts in *An. arabiensis *(A), *An. gambiae *molecular form M (M) and *An. gambiae *molecular form *S *(S) in experiment 1 to 4**. Boxes are 25^th ^to 75^th ^percentiles, lines 1.5 interquartile and dots outside values. Mean, standard deviation and median as well as Kruskall Wallis (K-W) and corresponding P value are given in the table.

The maximal number of oocyst was much greater in the *An. gambiae *S group (36), than in the two other groups (two in both). The distribution of oocyst number in each group is represented in Figure 2. The mean number of oocyst was variable across different groups in each experiment (Kruskall Wallis Chi^2 ^ranging from 0.0001 to 0.01), as well as globally for all experiments (p < 0.0001). When analysing all experiments globally, the number of oocyst was greater in the *An. gambiae *S group (1.72 ± 0.26) than in the *An. arabiensis *group (0.58 ± 0.04, Kruskall Wallis test p < 0.0001) and *An. gambiae *M group (0.64 ± 0.04, Kruskall Wallis test p < 0.0001). There was a significant although very weak correlation between the number of oocyst and the gametocytemia (Spearman test rho = -0.11, p = 0.01).

### Circumsporozoite protein rates

From the 600 fed mosquitoes used for CSP analysis, 311 survived for 14 days. Mortality rates were significantly higher in the *An. gambiae *S group (54.5%) than in the *An. arabiensis *group (41%, Fisher's exact test p = 0.005). The mortality of *An. gambiae *M group (49%) was not significantly different from the mortality in other groups (Fishers test p = 0.07 vs. *An. Arabiensis *and 0.16 vs. *An. gambiae *S group).

The parasite infection rates at sporozoite stage in each group are presented in Figure 1 panel B. In each experiment, there was a significant difference between groups (Pearson Chi^2 ^p ranging from 0.002 to 0.04) with *An. gambiae *S group being significantly more infected than *An. gambiae *M group in experiment 1, 3 and 4 (Fisher's test p = 0.001, 0.048 and 0.012 respectively) but not in experiment 2 (Fisher's test p = 0.101) and *An. gambiae *S group being significantly more infected than *An. arabiensis *group in experiment 1, 2 and 3 (Fisher's test p = 0.002, 0.003 and 0.017 respectively) but not in experiment 4 (Fisher's test p = 0.167). When analysed globally for all the four experiments, infection rate was higher in the *An. gambiae *S group (83.52%) than in the two other groups (50.85% for *An. arabiensis *and 50.98% for *An. gambiae *M respectively, Fisher's exact test p < 0.001 for each). On the other hand no significant difference was found between *An. arabiensis *and *An. gambiae *M groups (Fisher exact test p = 0.55).

## Discussion

This study is the first to evaluate the relative susceptibility of three major malaria vectors of the *An. gambiae *complex. It demonstrates that, in our experimental conditions, *An. gambiae *molecular form S is more susceptible than *An. gambiae *molecular form M and *An. arabiensis *to infection by *Plasmodium falciparum *at both oocyst and sporozoite stage.

Malaria transmission is known to depend on relationships that bind the pathogen, its invertebrate (vector) and vertebrate host (man). It is a complex phenomenon that implicates intrinsic factors associated with each actor as well as interactions between them [13]. The present study focused on the intrinsic capacity of the anopheline vector to be infected. This is the reason why it was performed on an *in vitro *model of mosquito infection that allowed a specific study of the ability of each vector to be infected [16]. Most extrinsic factors and parameters depending on the parasite and on the human host were controlled. As a matter of fact, in each experiment performed, the same parasite isolate was present in the same blood sample. The impact of environmental factors was also controlled since all the feeding experiments were conducted in uniform laboratory conditions. The results demonstrate that, these experimental conditions, the rate of infection was higher in molecular form S than in M form and *An. arabiensis *at both oocyst and sporozoite stages. The intensity of the infection was also higher in S form than M form and *An. arabiensis *as shown by higher mean number of oocyst. Although variations in infection rate and intensity were observed in the different experiments (representing the variability of parasitological and human factors), a similar difference between taxa was detected. This indicates that species and molecular forms belonging to *An. gambiae *complex did not display the same behaviour toward infection by *P. falciparum*. The difference observed between molecular forms M and S of *An. gambiae *and *An. arabiensis *was only related to their intrinsic susceptibility to *P. falciparum *infection. It is important to consider that although the method used for this study is the best available to evaluate susceptibility, it necessitates breeding mosquitoes in the insectary. Tested specimens belonged to the 3^rd ^generation and have, therefore, adapted to laboratory conditions. They may not be exactly representative of field populations.

During this study, mortality rate was different between species and forms. Although this parameter was not specifically studied, since all mosquitoes were potentially infected, mortality may have been influenced by the intensity of the infection. *Plasmodium *infection has been shown to reduce vector survival in laboratory conditions [18]. In this study, *An. gambiae *S form mosquitoes which were the most infected were also the less surviving at both oocyst and sporozoite stage. Even though many parameters have to be taken into consideration when evaluating malaria transmission in the field, the most efficient vector will be the one mostly highly infected and longest lived. The divergence of these two parameters among *An. gambiae *S and M forms could explain why dynamics studies of transmission have failed to demonstrated a difference between M and S forms [4,5]. The mechanisms responsible for parasite-induced mortality should be closely evaluated and taken into account in future anti-vectorial strategies.

The outcome of infection depends on the balance between the vector immune response, that aims to limit the infection [19,20], and the ability of the parasite to evade them [13]. The observed difference in susceptibility level among anopheles species to *Plasmodium *infection could be related to various levels of immune response of the vector. A variety of genes implicated in the immune response of the vector have been identified [21,22] and their allelic variants associated to relative refractoriness to infection [23]. A study on the repartition of the allelic variant of these gens in the different taxa inside *An. gambiae *complex could highlight their potential role in the difference susceptibility observed in this study. Beyond the M and S classification, the individual variation of susceptibility is a factor that should be investigated. The selection of resistant individuals may leads to refractory mechanisms at the molecular level, which could be exploited in the development of novel approaches to malaria control.

Susceptibility is known to increase with frequent and intense contact between parasite and vector because of evolution pressure of the parasite on the host [13]. This co-adaptation is also known to be dependent on the environment. Therefore, it would be interesting to study the relative susceptibility of molecular form M and S of *An. gambiae *from other areas or even their susceptibility to *Plasmodium *strains from distant regions. Interestingly, a large range of oocyst number was observed in experiment 2 in *An. gambiae *molecular form S. In the future, the relative variability of the susceptibility in the different taxa should be investigated.

## Conclusions

In the context of ecological speciation, this study is the first to demonstrate a difference between the molecular forms M and S of *An. gambiae *susceptibility for *P. falciparum *using an *in vitro *infection method. Infected in the same experimental conditions, molecular form S exhibited a higher susceptibility to infection by *P. falciparum *than molecular form M and *An. arabiensis*.

## Conflicts of interest

The authors declare that they have no competing interests.

## Authors' contributions

MON, JFT, CS and CB have equally contributed to the design, acquisition, analysis and interpretation of data. LK, AG and CS contributed to the conception of study and to the analysis of entomological data. AC performed field activities and molecular biology study. OF designed the study protocol. MON, CM and AC drafted the manuscript. CS provided the scientific supervision in Dielmo and Hanene. All authors read and approved the final manuscript.
